# Long-Term Analysis of Respiratory-Related Complications Following Gastrostomy Placement with or without Fundoplication in Neurologically Impaired Children: A Retrospective Cohort Study

**DOI:** 10.3390/children8010022

**Published:** 2021-01-04

**Authors:** Elisa Zambaiti, Calogero Virgone, Silvia Bisoffi, Roberta Stefanizzi, Francesco Fascetti Leon, Piergiorgio Gamba

**Affiliations:** 1 Division of Pediatric Surgery, Department Women’s and Children’s Health, University Hospital of Padua, 35121 Padua, Italy; elisa.zambaiti@hotmail.it (E.Z.); silvia.bisoffi@aopd.veneto.it (S.B.); francesco.fascettileon@unipd.it (F.F.L.); piergiorgio.gamba@unipd.it (P.G.); 2 Division of Pediatrics, Department Women’s and Children’s Health, University Hospital of Padua, 35121 Padua, Italy; roberta.stefanizzi@hotmail.it

**Keywords:** gastroesophageal reflux, neurological impairment, reflux-related complications

## Abstract

Gastrostomy placement is crucial in neurologically impaired (NI) children to ensure an adequate food intake and a safe route for drugs administration and to reduce the risk of primary aspiration. NI patents are more prone to gastroesophageal reflux. The association with fundoplication is deemed to reduce reflux-related respiratory complications. However, long-term benefits of this approach are not clear. We therefore aimed to compare long-term reflux-related respiratory complications of gastrostomy only (GO) to gastrostomy with fundoplication (GF). We retrospectively reviewed 145 consecutive NI children managed from 2008 to 2018. As long-term outcomes, we analyzed number and length of hospital admissions (Reflux-Related-Hospitalization, RRH) and emergency department accesses (Reflux-Related-Accesses, RRA) due to respiratory problems. Results were analyzed with appropriate statistical method. Median age at referral and at gastrostomy placement were 2.2 and 3.4 years (SD 5.6), respectively. Median follow-up was four years (range 1–12). Anti-reflux procedures were performed in 26/145 patients (18%); tracheotomy in 23/145 (16%). RRH following surgery showed lower number of admissions/year (0.32 vs. 1 for GO vs. GF, *p* < 0.005) and days hospitalization/year (3 vs. 13, *p* = 0.08) in GO compared to GF; RRA was similar (0.60 vs. 0.65, *p* = 0.43). Gastrostomy placement alone appeared not to be inferior to gastrostomy plus fundoplication with respect to long-term respiratory-related outcomes for NI children in our center.

## 1. Introduction

Children with neurologic impairment (NI) often experience feeding difficulties, which affect quality of life and contribute to long-term morbidity and eventually mortality due to failure to thrive, nutritional deficiency and respiratory insufficiency [1]. One of the main issues is the possible aspiration of pharyngoesophageal content due to either anomalous swallowing coordination or gastroesophageal reflux (GER), which may be defined as primary or secondary aspiration, respectively [2]. Repeated aspirations may cause acute and chronic lung disease and subsequent respiratory-related death [3]. In this population, nutrition and primary aspiration risks are often tackled with non-oral methods of food delivery, such as naso-gastric tube (NGT) or gastrostomy/gastrojejunostomy (GJ) placement [4], with the latter two being sometimes associated with an anti-reflux procedure (ARP) or even total esophago-gastric dissociation (TOGD) [5,6]. In NI children failure of ARP is reported high as 50% [7]. Although nutritional management is a crucial aspect for these patients, there is no univocal consensus on the approach to be applied [4]: this is mainly due to either lack of measured objective outcomes or to the reduced number of patients enrolled in each report.

Over the last 10 years, our Centre has preferred an approach consisting in minimal procedures, as a first line of treatment, and the use of more complex surgical interventions in selected cases or as second line of treatment, if required. This means that an ARP is rarely associated to a gastrostomy placement. The aim of this study was to compare long-term outcomes of these two options (gastrostomy only, GO, or gastrostomy with fundoplication, and GF), with a special focus on respiratory related problems.

## 2. Materials and Methods

### 2.1. Analyzed Outcomes

This is a retrospective cohort study. The descriptive analysis of the population included the surgical technique applied for gastrostomy placement, the length of the procedure, the number of post-operative doses of analgesics, the need of post-operative monitoring in an intensive care setting (ICU), the time needed to reach a complete enteral feeding, the length of hospital stay and the short term complications (within 30 days after the intervention).

The primary outcome measured for the long-term comparison between GO and GF groups, with the latter performed either during the same intervention or in two separate procedures, was the reflux-related hospitalization (RRH) modified from Srivastava et al. [6]. We included hospitalization for any respiratory distress, referred to GER, dysphagia, all types of pneumonia or asthma. We thus analyzed countable outcomes: number of admissions/year, days of hospitalization/year and days of hospitalization/admission, comparing pre-operative values with post-operative values and differences between groups. Each year of follow-up was counted as 365 days starting with the date of the intervention. We further evaluated reflux-related accesses (RRA) to the emergency department (ED) which did not lead to hospitalization.

Secondary long-term outcomes included late complications, occurring after at least 30 days following surgery and mortality for disease progression or concurrent conditions. Complications were ranked according to the modified Clavien–Dindo classification for surgical complications and considered major if of grade 3 or higher [8].

### 2.2. Study Population

Data from patients who received surgical consultation for feeding disorders at our Institution from January 2008 to December 2018 were retrospectively collected. We included all patients, aged less than 18 years at first consultation, with disorders related to NI, and who underwent a surgical procedure to specifically address this issue. Patients who did not receive a gastrostomy associated or not to a fundoplication, who received a GJ placement or TOGD, or whose surgical records were incomplete, were excluded from this study. In order to ensure an adequate follow-up, we excluded the most recent cases, while data collection for the included ones lasted until December 2019.

The reasons for surgical consultation varied, but most frequently, patients had dysphagia, malnutrition, symptoms of GER, such as recurrent aspiration pneumonia, grunting after meals, frequent regurgitation, and unexplained food refusal or food-related irritability. As per last ESPGHAN consensus [4], in this paper we defined NI an heterogeneous group of disorders, static or progressive, central or peripheral, which may affect an individual’s speech, motor skills, vision, memory, muscle actions and/or learning abilities. One of the reasons for NI is cerebral palsy, a group of permanent disorders of the development of movement and posture, which are attributed to disturbances that occurred in the developing fetal or infant brain [9]. In an attempt to objectify the level of NI, we sub-grouped the patients according to the Gross Motor Function Classification System (GMFCS), a 5-level clinical classification that describes the motor function on the basis of self-initiated movement abilities, ranging from 1 (can walk, run and jump with slightly limited speed, balance and coordination) to 5 (inability to sit, stand or maintain head against gravity). GMFCS was attributed at the first surgical consultation.

Variables potentially influencing the surgical strategy were collected, including the presence of comorbidities, type of pre-operative feeding (oral, enteral or by nasogastric tube, NGT) or presence of tracheostomy [10]. As diagnostic methods were used 24-h pH monitoring and radiologic studies like upper gastrointestinal tract barium swallow (UGI-BS) and video-fluoroscopy (VFS).

Both short- and long-term outcomes following the procedures were analyzed for each patient.

### 2.3. Statistical Analyses

Patients’ data were presented with descriptive statistics including frequencies, percentages, medians and ranges (as data were considered not normally distributed). Rate ratios (RR) and odds ratios (OR) were used to compare results and generate *p* values. Statistical analysis was conducted with the Mann–Whitney’s U test, the Fisher’s exact test and the 2-way ANOVA. Outcome were analyzed and results were displayed using GraphPad Prism 6 Software^®^.

## 3. Results

### 3.1. Overall Population

We identified 145 NI children who underwent surgical consultation for feeding disorders in the study period included. In this population, cerebral palsy was the main reason of NI (42 patients, 29%), followed by genetics (40 patients, 28%), metabolic disorders (24 patients, 17%), encephalopathy due to epilepsy (16 patients, 11%), acquired reasons, following trauma or cerebral infections (9 patients, 6%), mitochondrial disorders (5 patients, 3%), and miscellanea not otherwise classified (9 patients, 6%). Median age at first gastrointestinal assessment was 2.2 years (range 0 to 18); at that time, 40 were fed exclusively orally (27.6%), 7 had a mixed oral/NGT nutrition (4.8%), 68 had an enteral nutrition exclusively administered by NGT (46.9%), and 5 patients had parenteral nutrition as the main feeding source (3.5%). The nutrition modality was not known due to lack of data for 25 patients (17.2%). GMFCS was distributed as listed in Table 1.

Data on pre-operative diagnostic evaluation were available in 98 patients (67.6% of the entire population); 89 patients (91% of the available data) had performed tests for the assessment of either reflux, gastric emptying or swallowing ability; 9 patients did not perform any pre-operative evaluation. Eighty-one patients had performed a diagnostic test for reflux: 24-h pH monitoring was reported in 58 out of 81 patients, among those 29 diagnostic tests were positive for reflux (50% of the available data); 38 patients performed UGI-BS, 20 were positive for reflux (53% of the studies performed), 5 had delayed gastric emptying (13%). Forty-nine patients were diagnosed with reflux (55% of the evaluated patients), either by pH monitoring or UGI-BS, while 40 were negative (45%). Thirty-five patients have performed VFS before surgery; in 20 out of 35 (57%) the ability of swallow was impaired (Table 2).

Mean age at surgery was 3.4 years (range 9 to 18). Of 145 patients, 127 had an initial gastrostomy placement alone, and 18 had fundoplication done synchronously with the gastrostomy placement. Eight patients of the first group (GO) had fundoplication done as a second procedure (6%) at a mean time of 24 months after gastrostomy placement, and thus for long-term evaluation were transferred to GF group. None of those had hospital or ED admissions due to respiratory related issues in the time ranging from gastrostomy placement to fundoplication. Indication for fundoplication was mainly the presence of symptoms resistant to therapy.

Of the 127 patients in the GO group, the vast majority had the gastrostomy performed with the Gauderer–Ponsky percutaneous technique (91%) and only 12 had a laparoscopic-assisted PEG or a Stamm procedure (9%). All the 18 patients who had a concomitant fundoplication had the gastrostomy performed with either open surgery (5) or laparoscopic assistance (13). The further eight with the delayed fundoplication had the primary gastrostomy placed in six cases percutaneously, in one by open surgery and in one with laparoscopic assistance. The primary gastrostomy of the eight patients who received later the delayed fundoplication, was placed in six cases percutaneously, in one case by open surgery and in one case with laparoscopic assistance.

### 3.2. Short-Term Evaluation

The surgical procedure of gastrostomy placement took a median of 23 min (range 10 to 180) while the addition of the fundoplication increased the time to a median of 180 min (range 60 to 210).

Each patient had a median of 6 doses of analgesics administered in the post-operative period (range 0 to 48), with a median of 5 doses in the GO group and 14 doses in the GF group (*p* = 0.002, Mann–Whitney test). Overall, 28 patients (19%) needed a closer early post-operative monitoring, while 37 (26%) needed to be admitted in the ICU, mainly because of difficulties to be withdrawn from the ventilator support. The average ICU stay was 2 days.

Early complications were reported in 57 patients (39%), listed in Table 3. The distribution did not differ significantly neither between the different techniques used for gastrostomy placement nor in case of a concomitant ARP (RR 1.29 95%CI 0.81–2.03, OR 2.43 95%CI 0.69–9.24, *p* = ns, 2-way ANOVA).

Median stay in-hospital after the procedure was 5 days, ranging from 2 to 77. At discharge, 24 patients (17%) were fed exclusively with continuous nutrition, 22 (15%) had a combined feeding regime with bolus administration during the day and continuous nutrition overnight, 89 (61%) had bolus feeding only. Sixteen patients (11%) maintained some oral intake. Sixty-four patients reached full enteral feeding during the hospitalization in an average of 5 days (range 1 to 73), while 63 patients were discharged to reach full enteral feeding at home; those were discharged after a median of 4 days (range 1 to 27). The presence of NGT prior to gastrostomy placement did not correlate with a quicker achievement of full enteral feeding (RR 1.20 95%CI 0.88–1.63, OR 1.52 95%CI 0.75–3.09, *p* = ns, Fisher’s exact test).

### 3.3. Long-Term Outcomes

Considering long-term outcomes, 26 patients belonged to GF group while 119 to the GO group. In GF, 81% of the tested patients had a positive diagnostic test for reflux (21 positive patients/26 tested for reflux), while 43% in GO (27 positive/63 tested); in this context, the diagnostic tests for reflux have a predictive positive value for need for ARP of 43%, with a negative predictive value of 87%.

Overall, long-term follow-up of this cohort of patients is a median of 4 years, ranging from 1 to 12. At the time of the study, 48 patients were deceased. Excluding those, mean follow-up is 6 ± 3 years.

**Figure 1:**
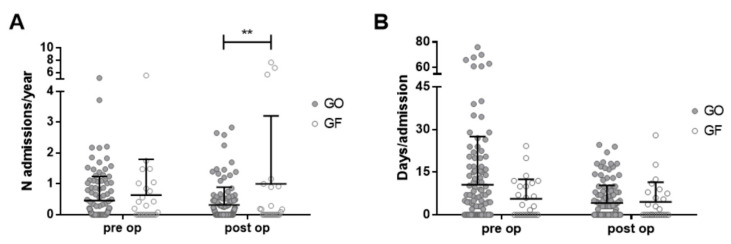
Long-term reflux-related hospitalization pre-operatively and post-operatively comparing gastrostomy only placement (GO) and gastrostomy plus fundoplication (GF). Bars for median and upper quartile. (**A**): Number of hospital admissions/years (*p* = 0.004, 2-way ANOVA). (**B**): Length of hospital stay/admission (*p* = 0.02, 2-way ANOVA). ** indicate that the difference between the two groups reached a statistical significance.

Number of hospital admissions for respiratory-related complications/year was significantly higher post-operatively in GF compared to GO, despite a comparable pre-operative value (post-operative 1.00 vs. 0.32, *p* = 0.004, two-way ANOVA, Figure 1A). Days of hospitalization/year are reduced post-operatively for GO (12/year vs. 3/year), while are doubled for GF (7/year vs. 13/year). However, the correlation between type of surgery and days of hospitalization/year did not reached significance (*p* = 0.08, two-way ANOVA).

Days of hospital stay per each admission are overall reduced after surgery (10 vs. 4, pre-operative vs. post-operative days/admissions) and for both GO and GF (11 vs. 4 and 6 vs. 5, respectively); the time effect is statistically significant (*p* = 0.02, two-way ANOVA, Figure 1B).

**Figure 2:**
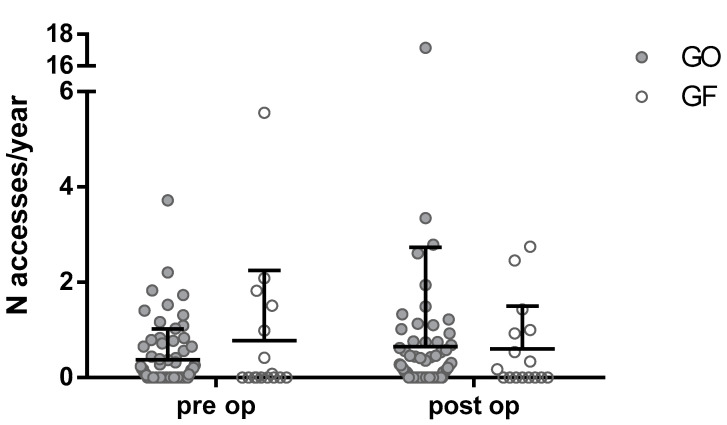
Number of Emergency Department admissions due to respiratory-related complications/years pre-operatively and post-operatively comparing gastrostomy only placement (GO) and gastrostomy plus fundoplication (GF). Bars for median and upper quartile. *p* = 0.43, 2-way ANOVA.

Overall, RRA to the ED were increased after surgery (0.06 vs. 0.17, pre-operative vs. post-operative accesses/year). Analyzing the type of surgery, GO placement increased RRA (0.37 vs. 0.65, pre-operative vs. post-operative accesses/year) while GF decreased it (0.78 vs. 0.60), despite the two-way ANOVA did not found any correlation between type of surgery and ED accesses (*p* = 0.43, Figure 2).

Twenty-two patients (15%) had major late complications, while thirty-five patients (24%) experienced minor complications (Table 3). As mentioned for short-term complications, the distribution did not differ significantly in case of a concomitant ARP (RR 1.25 95%CI 0.91–1.72, OR 2.80 95%CI 0.75–10.32, *p* = ns, two-way ANOVA).

During the data collection period, forty-eight patients were deceased: the main reason for that being either respiratory infection (14) or disease progression (26), no statistical differences were found between the two groups (RR 0.85 95%CI 0.74–0.97, OR 0.32 95%CI 0.10–1.02, GF vs. GO *p* = ns, Fisher’s exact test). Death occurred a median of 2.67 years following surgical intervention (range 1 month to 9 years). Of the patients deceased due to respiratory infection, the episode occurred at a median of 3.5 years following surgical procedure (range 1–7 years), in three cases the reason for the infection was a proven episode of ab ingestis. Further, in this subgroup, there was no s statistical differences between GF and GO (Fisher’s exact test). Comparative data on patients dead and alive at the time of the study showed no differences in rates of patients diagnosed with reflux (17 patients, 35% vs. 32 patients, 33%) or number of hospital admissions for reflux-related complications/year (0.55 vs. 0.37, *p* = 0.33 Mann–Whitney test).

## 4. Discussion

In this study, we report that gastrostomy placement alone may be not inferior to fundoplication in referral to long-term respiratory-related hospitalization indexes and accesses to the ED in a single center experience. As reported elsewhere, RRH is a composite measure of hospitalization for GER, dysphagia, aspiration pneumonia, and other types of pneumonia or asthma. Hospitalizations for these reasons are considered to likely represent complications of persisting GER disease: we included hospitalization for any respiratory distress because distinguishing aspiration/GER-associated disease from other types of pneumonia/asthma can be difficult in this sub-population and, if unrelated to GER, should anyway not introduce biases [11,12].

Compared to the available literature [7,12,13], our study tried to shed a light on a controversial topic: the advantage of an ARP to reduce respiratory impairment in the neuropathic child needing gastrostomy. Gastrostomy itself is a contributing factor in the decision for fundoplication in many centers; although open gastrostomy has been demonstrated to promote reflux [14], the minimally invasive techniques have been shown to not exacerbate reflux quantitatively or qualitatively in most children [15]. Moreover, different case series demonstrated that the need for fundoplication after gastrostomy placement ranges from 5 to 10% [5,16] and in our series we confirmed that 6% of patients needed an ARP after gastrostomy placement.

The indications to perform an ARP in this subgroup of patients is the most difficult issue in their management. We report an incidence of GER disease of 50%, while in literature it is reported to be around 20% [6]. However, the overall percentage of patients positive to reflux that undergoes an ARP is similarly of 25%. The decision whether to proceed with surgery in a patient with a positive history and a diagnosis of reflux may be controversial. In our center, we decided to perform fundoplication only in patients with symptoms resistant to medical therapy; this decision is also burdened by the different anesthesiology approach needed for the two procedures, and, namely, the use of deep sedation only in the majority of cases of gastrostomy placement, and the need for tracheal intubation for fundoplication. NI children are prone to adapt to the ventilation and it may be difficult to withdrawn them from it once adapted [17].

Rate of major short-term complications following gastrostomy placement has been recently reviewed [18]. The authors reported a rate of complications of the procedure in the included papers ranging from 0 to 15%; however, for the purpose of the study, they considered only those requiring reoperation within 30 days due to organ damage, excessive intra-abdominal leakage and fistula formation. The exclusion of other types of complications in their study, especially those related to infection, might account for the higher incidence in our series. The rates of complications observed in our series is similar to those previously described [19,20], although we might have lost some minor complications treated by local hospital and general practitioners.

Long-term outcomes are also controversial. Despite a large cohort study of children with NI demonstrated a reduction in hospitalizations for GER disease and mechanical ventilation after fundoplication, they could not demonstrate a reduction in hospitalization related to pneumonia [6]. A recent multicentric study from US failed to demonstrate a reduction in RRH the first year after concomitant gastrostomy placement and ARP [21]. Conversely, in another manuscript, fundoplication was found to be efficient in decreasing RRH in children who had preceding admissions related to reflux [12]. Referring to our data, the long-term morbidity in NI children who underwent any surgical procedure for dysphagia is reduced when considering pre-operative values, but the association of an ARP does not correlate with a better outcome compared to the gastrostomy placement only.

In most of these patients, the leading cause of death is often difficult to determine, as a progressive deterioration of overall clinical condition and the failure of many organs may occur. Notwithstanding the above, we may speculate that in our population mortality is not primarily due to reflux as both diagnosis of reflux and post-operative reflux-related admissions were similar to that of patients alive at the time of the study.

GJ feeding has been proven to cause more frequent hospitalizations due to admission for tube replacements and thus those children were excluded from our report. Notwithstanding, the same study reported an absence of differences in the hospital admissions for aspiration pneumonia or mortality [22], similar to the long-term results that we obtained comparing GO and GF.

Similar rates of respiratory infections (and hospitalizations) between the GO and the GF groups in the long-term should not be considered a surprising finding. The main reason of respiratory infections in this group of patients is indeed the poor oropharyngeal clearance of saliva and food, with subsequent high rate of primary aspiration [23], followed by gastroesophageal dysmotility, chronic constipation, scoliosis, or a predominantly supine position [13].

Another available alternative in NI patients is TOGD. Despite being initially used as a rescue procedure, TOGD is currently encouraged by many centers as a primary procedure in severely NI children [24]. Reviews of the technique quote a 20% risk of major complication following TOGD, with 5% mortality of the procedure [25]. In the experience of our and other centers, the decision to perform invasive procedures has to be confined to extremely selected cases as the advantages over gastrostomy placement only for these patients is limited [26].

The strength of this study includes the objectiveness of the measured parameters both for classification and outcomes that fails to provide subjective results depending on healthcare workers’ impression or parents’ satisfaction. The distribution of the GMFCS between the two groups, especially considering the severely impaired children with a grade 4–5, is equally divided. Moreover, most NI patients are centralized to our center for diagnosis and follow-up.

There are some limitations to this study. First, the retrospective nature of the analysis may have hampered the research of some parameters even if we have a digital recording software shared within the hospital that reduces the loss of information. In second instance, the selection of patients in the two groups may be biased: since the therapeutic approach was established case by case by our multidisciplinary team, the different initial characteristics that led to the decision, might have made the population of the two groups not completely homogeneous.

## 5. Conclusions

In NI children undergone a surgical procedure for feeding difficulties in our center, gastrostomy placement only seem to have long-term respiratory-related outcomes not inferior to gastrostomy with fundoplication. In our experience, fundoplication can be reserved to selected cases not responding to medical treatment.

## Figures and Tables

**Table 1:** Subgrouping of patients in the long term based on grade of neurological impairment according to Gross Motor Function Classification System (GMFSC).

GMFCS	All (145)	GO (119)	GF (26)
Grade 1, *n* (%)	7 (4.8)	5 (4.2)	2 (7.7)
Grade 2, *n* (%)	4 (2.8)	3 (2.5)	1 (3.8)
Grade 3, *n* (%)	8 (5.5)	6 (5)	2 (7.7)
Grade 4, *n* (%)	29 (20)	21 (17.7)	8 (30.8)
Grade 5, *n* (%)	88 (60.7)	75 (63)	13 (50)
unknown, *n* (%)	9 (6.2)	9 (7.6)	0 (0)

Legends. *n* = number; GO = gastrostomy only; GF = gastrostomy with fundoplication.

**Table 2:** Details on pre-operative diagnostic evaluation.

Diagnostic Test	Positive	Negative	Delayed Gastric Empting
24-h pH monitoring	29 (50)	29 (50)	
UGI-BS	20 (53)	13 (34)	5 (13)
VFS	20	15	

Legends. UGI-BS, upper gastrointestinal tract barium swallow; VFS, video-fluoroscopy. Percentage are provided between brackets.

**Table 3:** List of short and long-term complications. *n* = number.

Type of Complication	Short Term (*n* = 56)		Long Term (*n* = 57)	
	Minor (GF = 9, GO = 35)	Major (GF = 5, GO = 8)	Minor (GF = 5, GO = 30)	Major (GF = 7, GO = 15)
Bleeding	1 (GF)	1 (GF)	0	0
Organ damage	0	1 (GO)	0	0
Respiratory distress	0	3 (GF 2, GO 1)	0	5 (GF 2, GO 3)
Systemic infection/Fever	24 (GF 4, GO 20)	2 (GO 2)	1 (GO)	2 (GO 2)
Peritonitis/Bowel Obstruction	0	1 (GO)	0	4 (GF 1, GO 3)
Local infection/granuloma	5 (GF 1, GO 4)	0	15 (GF 3, GO 12)	0
Feeding difficulties/vomits	13 (GF 3, GO 10)	0	2 (GO)	0
Malfunctioning/Displacement	0	5 (GF 2, GO 3)	17 (GF 2, GO 15)	11 (GF 4, GO 7)
TOTAL (% of the patients)	43 (30%)	13 (9%)	35 (24%)	22 (15%)

## Data Availability

The data that support the findings of this study are available on request from the corresponding author. The data are not publicly available due to privacy or ethical restrictions.
